# Cultural interventions addressing disparities in the HIV prevention and treatment cascade among Black/African Americans: a scoping review

**DOI:** 10.1186/s12889-023-16658-9

**Published:** 2023-09-07

**Authors:** Shawin Vitsupakorn, Nia Pierce, Tiarney D. Ritchwood

**Affiliations:** 1 Trinity College of Arts and Sciences, Duke University, Durham, NC USA; 2 College of Humanities and Social Sciences, North Carolina State University, Raleigh, NC USA; 3 Division of Public Health Sciences, Wake Forest University School of Medicine, Winston-Salem, NC USA

**Keywords:** HIV/AIDS, Black/African American, Culture, Prevention, Treatment

## Abstract

Culture is an important determinant of HIV risk and protective behaviors; yet, we know little about how it is integrated in HIV interventions. This scoping review characterizes the integration of culture in HIV prevention and treatment interventions focused on Black/African Americans. We searched MEDLINE, PsycINFO, CINAHL, and Google Scholar for peer-reviewed manuscripts published between July 1, 2011, and June 28, 2021. Twenty-five interventions were identified, with 96% focused on prevention. Most (40%) targeted men who have sex with men or transgender women. Only three were grounded in cultural theory. Although all interventions were labeled “culturally based,” only two explicitly defined culture. Moreover, there was much diversity regarding the ways in which interventions integrated cultural elements, with some conflating race/ethnicity with culture. To improve uptake and HIV-related outcomes, interventions integrating culture are greatly needed. Additionally, HIV interventions purporting to be “culturally based” must include basic information to support rigor and reproducibility.

## Introduction

For forty years, HIV incidence and mortality in the United States (US) has declined due to innovations in biomedical HIV research, including the development of pre-exposure prophylaxis (PrEP) and antiretroviral therapy (ART), as well as the dissemination and implementation of behavioral interventions [1–6]. Despite this, engagement in the HIV prevention and treatment cascade remains suboptimal, particularly amongst those most burdened by the HIV epidemic, including Black/African Americans [7–9]. Rather than individual behaviors, the HIV burden experienced by Black/African Americans is deeply rooted in the racist history of the US; slavery, segregation, and mass incarceration have driven poverty and social disintegration in Black/African American communities, leaving them more vulnerable to syndemic threats, including HIV and substance use [10, 11]. Furthermore, these racist legacies have given rise to mistrust in health systems and providers, leading to lower engagement in the HIV prevention and treatment cascade and worse health outcomes [12, 13].

Among Black/African Americans, HIV has had a disproportionate impact on members of key populations, including men who have sex with men (MSM) and transgender women (TGW), youth, women, and other vulnerable groups [8]. Black/African American MSM, for example, comprise 26% of new diagnoses and 38% of diagnoses among all MSM [14]. While overall HIV prevalence appears to be decreasing or stabilizing among all Black/African Americans, MSM aged 25–34 years remain particularly vulnerable, as HIV prevalence in this age group rose 12% between 2014 and 2018 [15]. HIV among Black/African American MSM has been linked to a range of biomedical, social, and structural factors, including higher prevalence of sexually transmitted infections (STIs), less awareness of HIV status, lower adherence to and retention in HIV care, and racism, stigma, and discrimination [16–18].

Understanding the intersections between race, gender, sexuality, and socioeconomic status is essential to HIV interventions for Black/African American MSM. Moreover, it is important to acknowledge the limitations of the term “MSM,” as it homogenizes gender and sexual diversity (e.g., MSM who do not identify as gay, men who have sex with men and women (MSMW)) [19]. Data on TGW are often conflated with data on MSM despite their vastly different needs and experiences. From 2014 to 2018, HIV diagnoses increased by 9% among transgender people in the US, with some age groups (i.e., 25–34, 35–44, 45–54) seeing increases exceeding 30% [20]. Nearly half of TGW diagnosed with HIV in 2018 identified as Black/African American [20]. There are many factors associated with increased HIV risk among TGW, including depression, discrimination, homelessness or unstable housing, trauma, and lack of health insurance [21].

Black/African American youth (aged 13–24) have also been inequitably burdened by HIV; in 2018, they accounted for 21% of all HIV diagnoses [22, 23]. Compared to their adult counterparts, youth are less likely to test for HIV, know their HIV status, engage with care, and be virally suppressed than adults [24]. In 2018, 83% of HIV diagnoses in youth were among young MSM (YMSM), with more than half of these diagnoses among Black/African American YMSM—nearly twice the rate among Hispanic/Latino YMSM and over three times that among White YMSM [25]. Previous research has examined the role of culture in shaping HIV risk and behavior among youth, largely centering the influence of socialization on perception, communication, knowledge, and management of HIV [26–32].

​Compared to women of other racial and ethnic groups, Black/African American women in the US are disproportionately affected by HIV/AIDS [33]. In 2020, Black/African American women comprised 54% of HIV diagnoses among US women despite comprising only 13% of the female population [5]. This diagnosis rate is over four times that among Hispanic/Latina women and almost 11 times that among White women [5]. There are many socio-structural factors that contribute to this disparity, including poverty, lack of access to health care, stigma, higher rates of certain STIs, smaller sexual networks, and lack of awareness of HIV status [34].

Taken together, addressing racial inequities in HIV infection among Black/African Americans necessitates the development and advancement of multilevel interventions that not only integrate strategies to reduce individual risk but also address social and structural determinants of HIV risk [35, 36]. HIV researchers and practitioners have advocated for the integration of culture in HIV prevention and treatment interventions for decades [37–40]. There is wide acknowledgment that the days of “one-size-fits-all” approaches are over, and interventions designed to address mediating and moderating contextual factors, including systemic practices that enable disparities and inequities to flourish, are greatly needed [41–44]. Interventions that enable us to isolate the key components driving intervention effects are required, as they position us to determine which intervention works best for whom, when, where, and under which conditions.

Williams, Wyatt, and Wingood (2010), for example, argued that interventions addressing systemic barriers to equity in HIV-related outcomes and integrating socio-cultural determinants of sexual decision-making were greatly needed [42–46]. Yet, a decade later, there remains little understanding of the degree to which interventionists are integrating aspects of culture in HIV prevention and treatment interventions. HIV researchers and practitioners have long argued that the behavioral transmission of HIV occurs within a social context bound by culture—beliefs, values, norms, and traditions that are shared by members of a group and passed down over generations [40, 47–49]. As such, cultural scripts are believed to influence the norms and attitudes that govern engagement in HIV risk behaviors. Thus, when HIV prevention and intervention programs are grounded in culture, the programs may be more appealing, acceptable, and effective for the specific groups for whom they were developed. However, little empirical evidence exists to support this argument.

One systematic review concluded that, as of June 2011, no interventions were designed with the particular intention of reducing racial disparities in HIV acquisition [50]. Another review suggested that the vast majority of HIV prevention interventions in the US have neglected to consider culture, with many failing to either define or dissect it [40]. Such findings are highly problematic given that previous studies have shown that interventions with cultural elements were more effective than interventions that were not culturally grounded [37–39, 51].

The purpose of this scoping review is to synthesize the last decade (i.e., from 2011 to 2021) of research on HIV interventions specifically targeting Black/African American populations that are culturally based or informed. We extend previous reviews, which focused exclusively on HIV prevention [40, 50], to include interventions addressing the HIV treatment cascade, which includes a focus on HIV diagnosis, linkage to care, retention in care, adherence to ART, or viral suppression. In this review, we characterize the extent to which current US-based HIV prevention and treatment interventions stemming from randomized controlled trials (RCTs) targeting Black/African American communities integrate cultural beliefs, theories, measures, and curricula and report outcome-related data on whether such interventions were more effective than standard interventions that are not designed to integrate culture.

## Methods

We followed a five-stage approach to conducting a scoping review consisting of: 1) identifying a research question; 2) searching the literature for relevant articles; 3) article selection; 4) data charting; and 5) collating, summarizing, and reporting the results [52]. A scoping review is appropriate when the intention is to convey the breadth and depth of evidence on a selected topic where research is sparse, as well as elucidate gaps in the literature. In this review, we synthesize the evidence from RCTs on HIV prevention and treatment interventions for Black/African Americans that were explicitly described as being informed by culture. We employed a systematic approach to identifying studies to support replicability and reduce bias in study inclusion.

### Database search

Our search included studies published in peer-reviewed journals from July 1, 2011 to June 28, 2021. We searched four databases: PubMed, PsycINFO, CINAHL, and Google Scholar. Keywords included *HIV, AIDS, Black, African American, programs, services, and interventions*. A full description of the search terms is included in Table 1. To identify additional interventions, we reviewed the reference lists of included articles.

**Table 1:** Description of database search strategy

[ “HIV” OR (“human” AND “immunodeficiency” AND “virus”) OR (“human” AND “immuno-deficiency” AND “virus”) OR (“human” AND “immune-deficiency” AND “virus”) OR “AIDS” OR (“acquired” AND “immunodeficiency” AND “syndrome”) OR (“acquired” AND “immuno-deficiency” AND “syndrome”) OR (“acquired” AND “immune-deficiency” AND “syndrome”) OR “antiretroviral” OR “anti-retroviral” OR “anti-HIV”]
AND
[ (“African” AND “American”) OR (“Black” AND “American”) OR “African-American” OR “Black”]
AND
( “programs” OR “services” OR “interventions”)
AND
( “randomized controlled trial” OR “controlled clinical trial” OR “randomized” OR “randomised” OR “randomization” OR “randomisation” OR “placebo” OR “randomly” OR “trial” OR “groups”)
Limited to: 2011 – 2021

### Screening

All articles were imported into a reference manager software to support file sharing and data organization. The title of each article was screened independently by two co-authors, who determined whether a given article should be included in the review. After screening the first 500 titles, the authors met to clarify and refine the selection criteria. Once the two authors completed their individual title passes, they met to resolve conflicts. The two authors independently screened the included abstracts and full-text manuscripts, then met again to resolve conflicts.

### Selection criteria

Studies were included in this review if they: 1) were primary RCTs; 2) targeted Black/African Americans; 3) were conducted in the US; 4) had primary outcomes that were relevant to HIV prevention or treatment; and 5) explicitly mentioned that the intervention was culturally informed, based, or integrated. Studies were excluded from this review if they were: 1) pilots; 2) secondary analyses of RCTs; 3) reviews; 4) not peer-reviewed; 5) not available in full text; or 6) published before July 2011. This review focuses on RCTs due to their high rigor, ability to estimate intervention effectiveness, importance to intervention uptake in real-world settings, and value to funders.

### Data extraction

The following data were extracted from each study in the final sample: 1) authors’ names; 2) publication year; 3) sample size; 4) percentage of participants who identified as Black/African American; 5) whether researchers distinguished between Black and African American identity; 6) target population of the intervention; 7) sexual orientation and/or gender identity of participants; 8) part of the HIV cascade addressed; 9) theoretical foundations of the intervention; 10) how culture was integrated; 11) method of delivery; 12) who facilitated the intervention; 13) whether the intervention was delivered in a community setting; 14) level of community engagement in the research process; and 15) effectiveness of the intervention. The final sample was categorized by target population for further analysis.

## Results

**Fig. 1:**
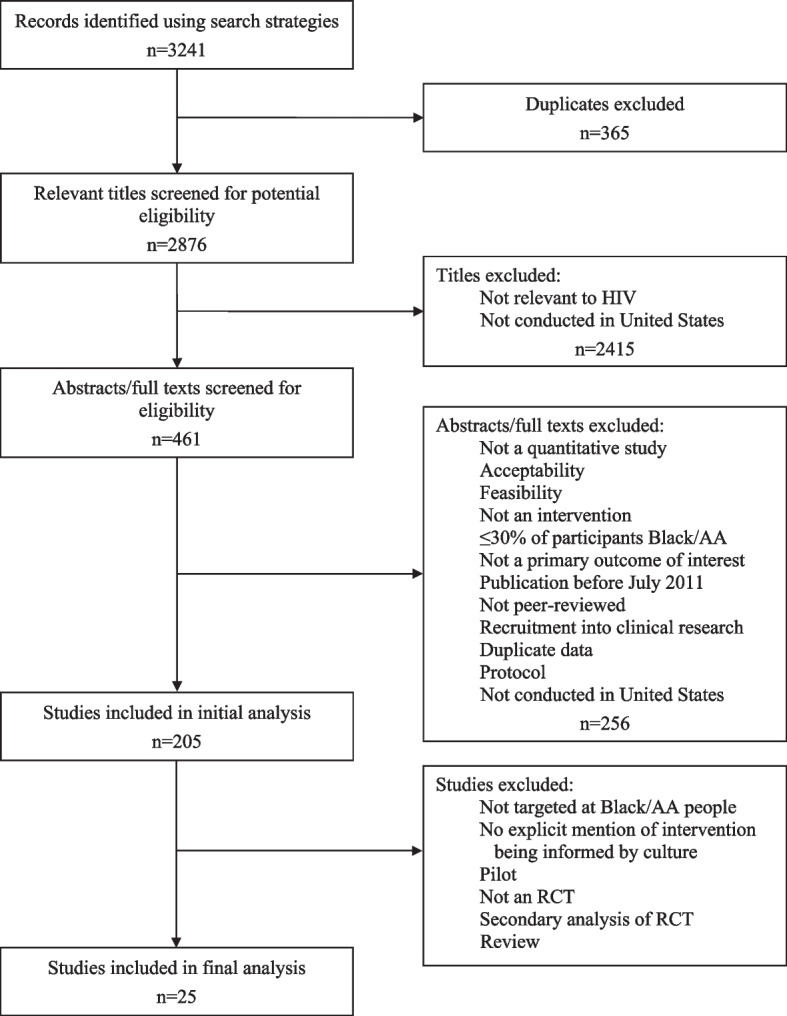
Results of literature search

Our search identified 2876 unique citations after removing duplicates, of which 205 underwent full-text review, and 25 met inclusion criteria (see Fig. 1).

### Study characteristics

Table 2 summarizes the study characteristics, including the participant demographics, cultural elements, and community engagement of the interventions. All studies were published between 2011 and 2021. For the youth studies, the mean age of participants ranged from 12.3 to 17.6 years. For the adult studies, the mean age of participants ranged from 20.6 to 48.5 years. The majority of studies (22/25) focused on HIV prevention, with the remaining studies (2/25) focusing on either prevention and testing or treatment engagement (1/25). Sample demographics ranged from 90 to 100% Black/African American participants. Fifteen of 25 interventions were delivered in person, seven of 25 were delivered digitally, and three were hybrid. We categorized the studies as targeting MSM and/or TGW (*n* = 10), youth (*n* = 7), women (*n* = 5), and other populations (*n* = 3). We describe the data from studies and relevant quality appraisal following these categories.

**Table 2:** Summary of intervention characteristics and treatment of culture

Author	Black/AA participants (%)	Distinction between Black and AA?	Target population	Sexual orientation and/or gender identity	Part of HIV cascade	Theoretical foundations	Integration of culture	Method of delivery	Who facilitated the intervention?	Delivered in community setting?
***MSM and/or TGW (n* = *10)***										
Fernandez et al. [53] (2016)	100	93% AA, 2% Black-Latino, 2% multiple Black identities, 3% other	MSMW (18 +)	84% bisexual, 7% gay/homosexual, 6% unsure/questioning/other, 3% straight/heterosexual	Prevention	Information-Motivation-Behavioral Skills Model	Negative stigma toward and discrimination against bisexuality; harmful stereotypes and media depiction of Black/AA men	Digital, real-time chat with facilitators	Not all ethnically matched; all had experience working with ethnic minority MSM	No, but developed in partnership with community advisory board
Arnold et al. [54–56] (2019)	100	No	MSMW (18 +)	Identifying as *not* gay/homosexual or bisexual	Prevention	Information-Motivation-Behavioral Skills Model	Masculinity and gender norms; secrecy and compartmentalization of sex with other men, desire not to be seen as gay; internalized, family, and church homophobia; discreet, one-on-one, non-judgmental intervention delivery; “men’s health” framing rather than “HIV prevention”	In-person	AA men	No, but developed via community-based participatory research
Hightow-Weidman et al. [57] (2019)	100	No	YMSM (18–30)	67% gay, 20% bisexual, 13% other	Prevention	Integrated Behavioral Model	Duality of being gay and Black; racism, homophobia, cultural/familial beliefs on masculinity; social isolation and rejection from Black community and church due to gay identity and/or HIV status; ball culture	Digital	N/A	No, but developed via formative research with community
Williams et al. [58] (2013)	100	No	MSMW living with HIV and history of childhood sexual abuse (18 +)	Identifying as *not* gay	Prevention	Social Cognitive Theory; Ecological Model	Triple minority identity (ethnic, sexual, living with HIV); the meaning of being an AA man; early socialization on gender and culture; adult experiences of being bisexual vs heterosexual within AA community; HIV stigma; recognizing stressors; learned strategies to cope with and regulate trauma	In-person	Ethnically matched men	No
Harawa et al. [59] (2013)	100	No	MSMW (18 +)	60% bisexual, 14% heterosexual, 12% gay/homosexual, 7% on the down low, 2% SGL, 5% none of the above/other	Prevention	Theory of Reasoned Action; Theory of Planned Behavior; Empowerment Theory; Critical Thinking and Cultural Affirmation Model	AA culture (e.g., collectivism, spirituality); historical events (e.g., slavery, Tuskegee, Million Man March); Ghanaian *Adinkra* symbols; racism; lack of identification with gay labels; gender expectations; importance of keeping sex with other men discreet	In-person	Ethnically matched men	Yes, and developed in partnership with community agencies and advisory board
Harawa et al. [60] (2020)	100	No	MSM (18 +)	64% homosexual, gay, or SGL	Prevention	Dynamic Social Impact Theory; Social Comparison Theory; Social Cognitive Theory	Intersectionality of HIV-related stigma, homophobia within Black community, and racism from the White community	In-person	Peer mentors (participants able to choose mentors)	No, but developed via formative research with community and in partnership with community advisory board
Harawa et al. [61] (2018)	100	No	Recently incarcerated MSMW (18 +)	71% bisexual, 17% gay/homosexual/SGL, 7% heterosexual, 5% other/none of the above	Prevention	Theory of Reasoned Action; Theory of Planned Behavior; Empowerment Theory; Critical Thinking and Cultural Affirmation Model	Adapted from MAALES (above, Harawa et al. (2013)); HIV risks and harm-reduction options in prisons and jails; challenges faced with reentry; HIV testing and stigma in custody settings	In-person	AA men	No, but developed with input from community advisory board
Jemmott et al. [62] (2015)	100	No	MSM (18 +)	41% bisexual 41% gay, 10% on the down low, 8% straight	Prevention	Social Cognitive Theory; Theory of Reasoned Action; Theory of Planned Behavior;	Importance of keeping sex with other men discreet	In-person	Not gender, racially, or ethnically matched	No, but developed via formative research with community
Herbst et al. [63–65] (2014)	100	68% AA, 17% Caribbean/West Indian, 11% Afro-Latino, 3% mixed ancestry, 1% African	MSM (18 +)	78% gay/homosexual, 18% bisexual, 3% unsure, 1% straight/heterosexual	Prevention	Social Cognitive Theory; Behavioral Skills Acquisition Model; Transtheoretical Model of Behavior Change; Decisional Balance Model	“Culture of Black MSM” session to help participants recognize how racism and homophobia are related to sexual and substance use risk behavior; duality of being Black and gay; relationships between STIs and HIV; familial, cultural, religious norms; sexual relationship dynamics common among Black MSM	In-person	Peers	Yes, and developed by CBOs serving Black MSM and a university-based HIV/STI prevention and training program via formative research with community
Frye et al. [66–69] (2021)	100	54% AA, 30% African/other, 7% Afro-Latino, 9% Caribbean	YMSM, YTGW (18–29)	60% gay/SGL/queer, 30% bisexual, 8% straight/heterosexual/other, 2% questioning/unsure	Prevention (Testing)	Socio-ecological Model; Empowerment Theory; Self-efficacy Theory; Social Support Theory; Motivational Interviewing Theory	Intervention labeled “culturally-congruent,” but culture not defined	In-person	Peers	No, but developed via formative research with community
***Youth (n* = *7)***										
Klein et al. [70, 71] (2011)	100	No	Female youth (14–18)	Sample characteristics not described; intervention designed for heterosexual youth	Prevention	Social Cognitive Theory; Theory of Gender and Power	Adapted from SISTA [72], WiLLOW [73], and SIHLE [74]; culture defined as that of “young AA females”; cultivating gender and cultural pride through readings of poetry by AA women, AA art, learning about strong AA female role models; tailored language; games; AA teenage girls to perform as “Sistas” in video clips	Digital	N/A	No, but developed alongside team of young AA girls
DiClemente et al. [75] (2014)	100	No	Female youth (14–20)	Sample characteristics not described	Prevention	N/A	Intervention labeled “culturally appropriate,” but culture not defined	Digital	Phone counseling delivered by AA female health educators	No
Hadley et al. [76] (2016)	100	24% with an additional racial identity 5% with Hispanic/Latino ethnic identity	Youth (13–18) and caregivers	Sample characteristics not described	Prevention	Social Learning Theory	Adapted from STYLE [77]; DVD as a culturally sensitive technology for families; content tailored to youth; all featured actors were 16–18 (“near-peers”); different DVDs for youth and caregivers (e.g., hip-hop music for youth, parenting guidance for caregivers); developed with minority-owned health communications company specializing in culturally relevant products for urban and ethnic audiences	Digital	N/A	No
Jemmott et al. [78] (2020)	90	No	Youth (11–14) and caregivers	Sample characteristics not described	Prevention	Social Cognitive Theory; Theory of Planned Behavior	Video content labeled as “culturally appropriate,” but culture not defined	Hybrid^a^	AA adults	Yes, and developed with input from community advisory board
Sznitman et al. [49, 79, 80] (2011)	100	No	Urban youth (14–17)	Sample characteristics not described	Prevention	Social Cognitive Theory of Mass Communication; Social Cognitive Theory	Broadly defined culture as “values, beliefs, norms, and behaviors shared by a group”; in-depth discussion of culture; acknowledgement of culture as dynamic and non-uniform; recognition of “skilled, expressive, and persuasive speech” as valued part of AA oral culture and dramatic formats as more persuasive than lecturing; Afrocentric vernacular; using ideas of Black masculinity that center on mutual respect and respectability rather than “sex as a conquest”; social pressures against delaying sex and using condoms	Digital (i.e., radio, TV)	Messages written by advertising professionals experienced in reaching AA youth	No, but developed via formative research with community and in partnership with community advisory board
Donenberg et al. [81] (2020)	100	No	Female youth (14–18) and their female caregivers	Sample characteristics not described	Prevention	Social-Personal Framework; Social Learning Theory*; Social Cognitive Theory*; Theory of Gender and Power	Adapted from SISTA [72], SIHLE [74], and STYLE [77]; “labor and power gender divisions”; gender-specific standards for which sexual behaviors are appropriate in heterosexual relationships; ethnic and gender empowerment	In-person	Black/AA women experienced at working with youth in health settings	No, but developed with input from community advisory board
DiClemente et al. [82, 83] (2014)	100	No	Female youth in juvenile justice system (13–17)	Sample characteristics not described	Prevention	ADAPT-ITT Model [84]; Social Cognitive Theory; Theory of Gender and Power	Adapted from HORIZONS [85]; intervention labeled as “culturally appropriate,” but culture not defined; ethnic and gender empowerment	In-person	AA female health educators	No, but developed with input from teen and community advisory boards
***Women (n* = *5)***										
Billings et al. [86] (2015)	100	No	High-risk women (18–50)	Sample characteristics not described	Prevention	Empowerment Theory	Message of intervention reinforced via unscripted videos of AA women describing importance of self-confidence and pride in being a Black/AA woman; intersectionality as a Black woman; framing condom use as a goal for women (rather than a behavior for men)	Digital	Black/AA women	No
Gilbert et al. [87] (2021)	100	No, but 23% identified as Latinx	Women with drug history in CSPs (18 +)	66% heterosexual, 31% bisexual, 3% other	Prevention	Empowerment Theory; Social Cognitive Theory	Raises awareness of structural racism rooted in slavery and historical responses of resilience among Black/AA women; “explicit focus on structural racism along with its novel hybrid group format led by Black female CSP staff and a computerized individualized tool with Black women characters promoted effective cultural tailoring of content”; Afrocentric themes of historical trauma and resiliency	Hybrid	Black/AA women (counselors or case managers at CSPs)	Yes, and developed via community-based participatory research
Wingood et al. [88] (2013)	100	No	Women (18–29)	Sample characteristics not described	Prevention	Social Cognitive Theory; Theory of Gender and Power	“Valuing one’s body, perceiving one’s body as a temple” described as “culturally appropriate connotation”	In-person	Black/AA female health educators	No
Painter et al. [89] (2014)	100	No	Heterosexual women (18 +)	Sample characteristics not described	Prevention (Testing)	Health Belief Model; Transtheoretical Model of Health Behavior Change; Social Cognitive Theory	Shared cultural aspects of Black/AA women’s experiences that shape vulnerability to HIV (e.g., intersectionality of being a Black woman in the South); Black/AA women’s collective wisdom and lived experiences	In-person	Black/AA women	Yes, developed out of a CBO
DiClemente et al. [90] (2021)	100	No	Women (18–24)	Sample characteristics not described	Prevention	Social Cognitive Theory	Emphasizing gender and ethnic pride	Hybrid	Black/AA female health educators	Yes, and developed with input from community advisory board
***Others (n* = *3)***										
Bogart et al. [91] (2017)	100	No, but 6.5% identified as Latino	Adults living with HIV (18 +)	36.3% straight, 42.8% gay man, 1.9% lesbian, 13.5% bisexual man, 1.4% bisexual woman, 1.4% not sure, 2.8% other	Retention in/ Adherence to care/Linkage to treatment	Social-Ecological Theory; Information-Motivation-Behavioral Skills Model	Survival mechanisms historically used by Black/AA people to cope with oppression: (1) adaptive duality (2) collectivism, (3) indirect communication, and (4) mistrust of outsiders; defined “culturally congruent” as customized to fit values, beliefs, traditions, and practices; gives examples of cultural and social determinants of health behavior of AAs as medical mistrust, HIV misconceptions, and experiences with discrimination; recognizes cultural issues associated with treatment nonadherence (e.g., medical mistrust, discrimination, internalized stigma)	In-person	Black/AA peer counselors	Yes, developed with community advisory board and CBO
Stewart et al. [92] (2017)	100	No	Rural cocaine users (18 +)	Sample characteristics not described	Prevention	Social Cognitive Theory; Transtheoretical Model of Change	Culturally adaptation included revising intervention scripts to reflect rural drug setting, same-gender group sessions, restricting attendance to participants only, and hosting follow-ups in study offices rather than on the street; hygiene, condoms, and domestic violence identified as topics unique to community; engaging “book smarts and street smarts” by using both scientific and street terms and professional and lay facilitators	In-person	Black/AA health educator and peer leader	Yes, and developed via community-based participatory research
Yancey et al. [93] (2012)	100	No	Heterosexual men and women (18–44)	Sample characteristics not described	Prevention	*NTU* (Africentric) conceptual framework; Theory of Gender and Power; Nguzo Saba	Inspired by African cultural principles of Umoja-unity, Kujichagulia-self-determination, Ujima-collective work and responsibility, Ujama-cooperative economics, Niapurpose, Kuumba-creativity, and Imanifaith; entire session dedicated to intersection of culture- and gender-related issues; gender and culture stressed throughout the intervention	In-person	Co-ed facilitator teams	Yes, and developed via community-based participatory research

*Abbreviations: AA* African American, *MSM* men who have sex with men, *TGW* Transgender women, *MSMW* men who have sex with men and women, *YMSM* young men who have sex with men, *YTGW* young transgender women, *SGL* same gender loving, *STI* sexually transmitted infection, *CBO* community-based organization, *CSP* community supervision program

^a^“Hybrid” delivery method refers to a combination of in-person and digital components

#### Men who have sex with men (MSM) and transgender women (TGW)

We identified 10 interventions for Black/African American MSM and/or TGW. Of these, five (50%) focused on MSMW, four (40%) on MSM, and one (10%) on both MSM and/or TGW. All 10 interventions focused on the HIV prevention cascade, with one [66] focusing on HIV testing and the remainder emphasizing reducing HIV risk and increasing engagement in protective behaviors. Eight interventions (80%) were delivered in person, with the remaining two interventions (20%) delivered digitally. Six interventions (60%) demonstrated effectiveness, with notable differences between the intervention and control groups. Specifically, compared to the standard of care or control groups, findings suggested that more interventions that integrated culture in some form reported greater effectiveness than those that did not integrate culture.

Three interventions (30%) distinguished between Black/African Americans at baseline, asking participants for their ethnicity (e.g., Afro-Latino, Caribbean). However, none addressed this diasporic diversity in their intervention design. All 10 interventions asked for self-reported sexuality, with two targeting MSMW who did not identify as gay or bisexual. All were grounded in theory, with two (20%) using the Critical Thinking and Cultural Affirmation Model, developed by and for Black/African Americans. Eight interventions (80%) explored how different identities (e.g., Black/African American, gay) intersected with each other. Seven (70%) employed Black/African American men as facilitators, with three (30%) being peer-based. Nine (90%) were developed through community-engaged research. One intervention, while labeled “culturally congruent,” did not include an explanation of how culture was incorporated into the design. None of the interventions explicitly defined culture.

Most of the interventions addressed the dual experience of being a Black/African American man who has sex with other men, including facing racism from non-Black people and homophobia/biphobia within the Black/African American community. One intervention, the *Bruthas Project* [54], made a concerted effort to accommodate the culture of MSMW who did not identify as gay or bisexual. Data from their formative research suggested that researchers often fail to recruit MSMW for HIV interventions that explicitly target MSM, as many preferred to keep their sexual activity with other men private or did not want to participate in a program focused on HIV. Equipped with this understanding, counseling was delivered one-on-one at discreet locations, and the intervention was branded as “Black Men’s Health,” rather than an HIV prevention program [55]. Another intervention, “MAALES” [59, 94], while also for MSMW, was delivered in small group settings. Rooted in the Afrocentric theory, MAALES featured elements such as a *Fihankra* safe space and Ashe affirmation circle, all designed to leverage African cultural practices.

#### Youth

We identified seven interventions targeting Black/African American youth. Of the seven interventions, four (57%) focused on female youth; three (43%) on parent-caregiver dyads, with one of the three designed for mothers and their daughters; one (14%) on youth in the juvenile justice system; and one (14%) on youth from urban settings. All seven focused on the HIV prevention cascade. Two (29%) interventions were delivered in person, four (57%) were delivered digitally, and one (14%) used a hybrid model. Only one intervention (14%) was delivered in community settings. All seven interventions showed evidence of effectiveness, with significant differences between the intervention and control groups.

None of the interventions in our sample made a distinction between Black and African American identity. One, however, asked participants if they identified with any additional racial and/or ethnic identities. Although one intervention was designed for heterosexual-identifying youth, none of the studies asked participants to self-report their sexual orientation. Six (86%) interventions were grounded in theory: five of the six were based on Social Cognitive Theory, three on the Theory of Gender and Power, and two on Social Learning Theory. None, however, used theory developed specifically by or for Black/African Americans. Three interventions (43%) considered the intersection of identities (e.g., being both Black/African American and a youth), with two focusing on gender and ethnic empowerment among female youth. Four of the interventions (57%) employed Black/African Americans as facilitators, with three matching by gender (i.e., Black/African American women for young girls). Four (57%) interventions were developed in collaboration with the community. Lastly, three interventions (43%) were labeled as culturally based but did not explicitly state how culture was integrated.

Notably, culture was explicitly defined in only one intervention, Project iMPACCS [80]. In that study, the researchers defined culture as the “values, beliefs, norms, and behaviors shared by a group” and described it to be dynamic, complex, and non-uniform; culture is comprised of “surface” and “deep” structures, both of which are necessary to ensure the feasibility, acceptability, and effectiveness of interventions [49, 95]. Formative research informed the design of Project iMPACCS, which opted for dramatic messaging that would appeal more to younger audiences. Additionally, the researchers recognized the significance of expressive language and African-American Vernacular English to Black/African American oral culture [79].

#### Women

 We identified five interventions for Black/African American women. Of these, one (20%) focused on women at high risk, with “high-risk” defined by Billings and colleagues [86] as having “multiple male sexual partners in the past 2 months” or “inconsistent condom use over that same time frame with a man who was HIV positive, was an injection drug user, had concurrent sexual partners, or had not been tested for HIV since the onset of the sexual relationship.” Two interventions [87, 90] (40%) focused on women who use substances. All five interventions targeted HIV prevention, with one focusing on HIV testing. Among the interventions, two (40%) used a hybrid model, and one (20%) was delivered digitally. The remaining studies were delivered within traditional and community clinics. For example, DiClemente and colleagues [90] used group motivational therapy at local clinics, and Painter and colleagues [89] presented single-session community modules also in clinics. All studies reported similar results, such as improvements in condom use frequency, HIV knowledge, sexual communication, sexual refusal, and/or safe sex initiation.

All included studies that focused on women reported that their interventions were culturally tailored, largely defined as having racial representation among some study staff members. Frequently, interventions had Black/African American women facilitate intervention sessions. Moreover, all studies described their intervention tools and materials as being representative of Black/African American women. For example, Billings and colleagues [86] developed a multimedia intervention to teach Black/African American women effective ways to refuse sex while also reducing the risk of intimate partner violence. They described the intervention as promoting gender empowerment and feelings of positive racial identity. To accomplish this, the researchers recruited Black/African American women to record unscripted videos personally expressing why they feel proud of their racial and gender identity. Gilbert and colleagues [87] adapted an intervention originally designed to reduce STI incidence and condomless sex among Black/African American women in community supervision programs. They described their process of cultural adaptation as incorporating information about the historical contexts of racism, its impact on Black/African American women, and how Black/African American women have stayed resilient in response. They also employed Black/African American women as facilitators of the in-person sessions, with all digital sessions featuring Black/African American female characters. This RCT was the first to show positive effects of a culturally tailored intervention on reducing STI incidence and condomless sex among Black/African American women in community supervision programs [87].

#### Other populations

​We identified three studies that focused on other key populations, including people who use alcohol or other substances. One intervention, for example, focused on promoting HIV prevention strategies among rural residents who use cocaine [92]. The authors reported that the intervention was culturally tailored, such that Black/African Americans who also had histories of cocaine use served as peer leaders, and they designed the intervention to leverage community engagement to build social capital and trust between facilitators and the participants.

Another HIV prevention intervention, for example, was designed to target beliefs, attitudes, and behaviors associated with engagement in HIV risk behaviors, HIV-related stigma, and the impact of media on risk behavior [93]. In this study, they found that using this culturally tailored intervention promoted overall HIV risk knowledge, safer sex peer norms and conversations, and a significant decrease in risky sexual behavior.

Of the three interventions targeting other populations, only one study provided an explicit definition of “culture.” Bogart and colleagues [91] defined a “culturally congruent” intervention for Black/African Americans to be one that is “customized to fit their values, beliefs, traditions, and practices.”

## Discussion

This scoping review described trends in HIV prevention and treatment intervention research targeting Black/African Americans in the US published in the past decade (2011–2021), where authors reported that their interventions were culturally informed or culturally based. We identified 25 unique interventions that explicitly mentioned that culture had informed their intervention design. It was notable that only one study in our sample [91] focused on the HIV treatment cascade, highlighting a persistent gap in culturally based interventions specifically designed to engage Black/African Americans in HIV care. It is also important to note that, although all 25 interventions that we evaluated were described as being “culturally based” or informed by a culturally based framework, none of the articles included in this review both (1) operationally defined what was meant by “culturally based” and (2) reported criteria on what parameters were applied to qualify their interventions as such. In fact, only two studies [80, 91] explicitly defined culture. Without reporting this information, interventionists are limited in their ability to critically evaluate the rigor of intervention research or reproduce it in similar populations. Moreover, practitioners seeking to utilize these interventions within their practices or communities lack critical information that would enable them to determine whether a particular intervention is appropriate for their target population.

Despite calls for the integration of culture in interventions for Black/African Americans, results of our scoping review indicate that few have done so. Moreover, the way in which culture is operationalized and integrated throughout the intervention is often unclear. The implementation of explicitly defined cultural concepts is possible; a previous review focusing on Latino populations [96], for example, showed that HIV interventions that were designed to address *machismo* were associated with decreased sexual risk-taking. More attention to methodological approaches would enable investigators to identify aspects of culture that may influence HIV risk, and establishing operational definitions allowing for comparisons across trials may support the advancement of the science in this area.

Though not always explicitly stated, interventionists seemed to rely upon formative research (e.g., focus groups) or co-design processes to infuse culture within their interventions. In our review, for example, most studies (*n* = 20 of 25) engaged Black/African Americans residing in targeted communities in the design process by consulting a community advisory board or conducting formative research. While these may be valid approaches, it is unclear whether they are sufficient for ascertaining *cultural* values. Since the quality of these interactions and formative data likely vary significantly and few authors reported detailed information regarding their approaches to obtaining specific information about culture, it is difficult to determine best practices in collecting such data. Black/African Americans have a complex history in the US, and many may be unable to articulate the influence of *culture* on their behaviors, as much information regarding the reasons for certain behaviors were lost during enslavement for those with strong generational history in the country. Moreover, there is much diversity within this group, with many having closer ties to their cultural histories due to more recent immigration statuses. As such, it is unclear how one might develop a culturally based intervention without determining predominant cultural beliefs and practices in a specific community, as it may be inappropriate to focus on racial similarities alone.

A more nuanced approach to developing culturally based interventions may be necessary, with interventions including information regarding how implementation may differ for Black/African Americans from certain cultural backgrounds and regions. This information could be included in a protocol as “additional cultural considerations” to highlight the diversity within this group and how interventions may need to be adapted accordingly. Previous research highlighted the need for HIV interventions to integrate open discussions on systemic racism [97]. For instance, conspiracy beliefs regarding HIV must not be met with judgment but rather an understanding of the oppression faced by Black/African Americans, which requires exploration via open-ended questions and affirmations.

This scoping review has several strengths. First, we extended the review beyond HIV prevention to include treatment interventions targeting Black/African Americans. This highlighted a persistent gap in the literature, namely that culturally informed interventions focused on the HIV treatment cascade remain limited. Secondly, unlike previous reviews that limited studies to those conducted in facilities [50], we included those conducted in community settings as well. This decision proved insightful, as several interventions in the final sample were community-based. Lastly, although this was a scoping review, we employed a systematic approach to identifying studies for our sample to support replicability. Still, this review has several limitations. We did not contact researchers whose publications did not explain how culture was integrated in their intervention. Furthermore, since this was not a systematic review, we did not perform any quality or bias assessments, as our purpose in conducting a scoping rather than systematic review was to identify gaps in the literature to inform future research directions. Additionally, this study focused only on RCTs. As such, it is possible that important studies were excluded from our review, limiting the potential for generalizability. However, the primary goal of this scoping review was to highlight current knowledge and gaps in the field that could inform future directions. In the absence of a meta-analysis, we were not able to evaluate the effectiveness of cultural interventions on HIV-related outcomes among Black/African Americans.

## Conclusions

Findings from this scoping review reveal continued gaps in the literature regarding the integration and definition of culture in interventions targeting Black/African Americans. Even among studies that incorporated culture, the way in which culture was defined and incorporated within the interventions was often unclear. Our findings highlight a need for greater clarity and transparency regarding the way in which investigators conceptualize “culturally based interventions.” This could support the development and implementation of interventions that intervene at the socio-cultural level, which may redress the upstream factors driving HIV risk and infection that disproportionately impact Black/African American communities.

## Data Availability

All data generated or analyzed during this study are included in this published article and data files are available from the corresponding author upon reasonable request.
